# Influence of work-related stress on patient safety culture among nurses in a tertiary hospital: a cross-sectional study

**DOI:** 10.1186/s12912-023-01695-x

**Published:** 2024-01-31

**Authors:** Mohammed Mohammed Sani, Yahaya Jafaru, Daniel Opotamutale Ashipala, Abubakar Kalgo Sahabi

**Affiliations:** 1 Department of Nursing Science, College of Health Sciences, Federal University Birnin-Kebbi, Birnin-Kebbi, Kebbi State Nigeria; 2 School of Nursing and Public Health, Faculty of Health Sciences and Veterinary Medicine, University of Namibia, Rundu, Rundu, Namibia

**Keywords:** Influence, Nurses, Patient safety, Stress, Tertiary hospital

## Abstract

**Background:**

One of the global issues facing the nursing profession is work-related stress because it interferes with care quality and organisational competency. These kinds of stressful situations can cause damage to the mental ability of the affected individual resulting in low job productivity. In a Nigerian healthcare setting, patient safety is under-researched.

**Aim:**

This study aimed to assess the influence of work-related stress on patient safety culture among nurses in a tertiary hospital.

**Materials and methods:**

The study adopted a descriptive cross-sectional survey. The Population of the study was nurses who are currently serving as employees at Federal Medical Center Birnin-Kebbi. Proportional and systematic sampling methods were used in the selection of the sample of the study. The tools used for this study were adapted Hospital Survey on Patient Safety (HSOPS) and Nurses’ Occupational Stressor Scale. Ethical approval was obtained from the research ethical committee of the hospital.

**Results:**

The moderate stress experience was having the highest percentage (45.0%). The highest percentage of the nurses (69.9%) practised a moderate safety culture. There were weak or very weak significant negative correlations (*P* < 0.01) between patient safety culture practices and occupational stress across all the subscales of the nurses’ occupational stressors scale except in the occupational hazards subscale in which there was extremely weak and non-significant negative correlation. Work–family conflict was a significant predictor of patient safety culture, t (208) = -2.341, *P* < 0.05. Difficulty in taking leave was a significant predictor of patient safety culture, t (208) = -2.190, *P* < 0.05.

**Conclusion:**

There was a significant negative correlation between stress and safety practice which implies that as stress increased safety practice decreased. These study findings can be used to develop ongoing strategies and targeted interventions in addressing work-related stress.

## Introduction

Stress denotes a situation in which perceived environmental demands exceed the coping ability of an individual [1]. Work-related stress is found in one’s working environment and is interchangeably used with occupational stress or job stress [2]. Veda and Roy defined work-related stress as “a situation wherein job-related factors interact with an employee, changing his/her psychological and physiological condition in a way that the person is forced to deviate from normal functioning” [[3] P.68]. The authors identified common symptoms of stress as emotional imbalance, uncooperative attitudes, inability to cope, sleeping disturbances, easy irritability, excessive worrying, and difficulty in relaxing. These factors predispose persons to decreased work efficiency [3]. Alamri and Almazan [4] opined that stress is of greater concern for health professionals, notably nurses who are at the frontline in facing a high level of stress in the clinical environment.

Globally, a critical element of healthcare quality that has been a public concern across the healthcare system is patient safety, and the culture of safety among healthcare professionals is among the major pillars of promoting patient safety [5]. Patient safety is the act of error and adverse effect prevention when rendering healthcare services to patients [6]. It aims to prevent harm to patients [7]. Patient safety culture is the individual and organisation’s values, attitudes, perceptions, competencies, and patterns of behaviour that determine healthcare system safety culture and safety management [8].

One of the global issues facing the nursing profession is work-related stress [9]. This is because it interferes with care quality and organisational competency [10]. The healthcare industry is believed to be among the most hectic industries [11], with clinical nurses being confronted with stressful conditions such as handling conditions, not responding to treatment and the death of a patient [12]. Ezenwaji et al. [13] asserted that encountering patients with severe illnesses and patient death makes the nursing profession among the most stressful professions. These kinds of stressful situations can cause damage to the mental ability of the affected individual, resulting in low job productivity [14, 15]. This means that the caring behaviour of nurses can be affected by stress [16].

Working as a nurse is stressful, and stress can adversely affect nurses’ physical and mental well-being as well as their professional performance [17]. Nurses’ quality of life may be affected by job stress, which could affect the quality of nursing care [18]. The better the nurses’ quality of life the higher the effectiveness of nursing care [19]. However, approximately one-third of the global nurses face a high level of hospital practice-related stress [20]. Some studies revealed that more than half of nurses encountered occupational stress [21, 22]. In a similar study from Nigeria about four-fifths of nurses reported moderate job stress, while 10.62% reported job stress to be severe [23]. A study in Ethiopia found the prevalence of work-related stress among nurses to be greater than half, indicating nurses’ workload as the most frequently stressful subscale reported by nurses. This is followed by the incidence of dying and death [24]. The effects of these stressful situations may affect the patient safety culture practice among nurses.

It has been asserted that 1 in 300 patients receiving healthcare is at risk of death from preventable medical accidents. As many as 4 out of 10 patients are harmed while receiving healthcare across healthcare settings worldwide, and up to 80% of this harm is preventable [25]. The Director of Patient Safety Africa described the challenge in Nigeria as follows: “Citizens and patients are dying in hundreds because of lack of access to care or from poor care. Healthcare workers are adding to the problems rather than solving them” [26]. However, globally, few studies have investigated the influence of work-related stress on patient safety culture among nurses [20, 27–29]. There are studies on patient safety culture [30–33] and work-related stress [34–38] among nurses in Nigeria. Nevertheless, it is the notable study by Kaware et al. [39] that tried to relate patient safety culture with some aspect of work-related stress (working hours and staffing).

Evaluating the current patient safety culture enables the identification of the safety behaviours of hospital staff and the areas that need improvements [40]. Work-related stress is considered among the most common mediators that make healthcare professionals including nurses leave the profession [41]. Researching work-related concerns among nurses could be important in identifying how the rate of occupational burnout and job dissatisfaction could be reduced. Thus, it is an essential requirement to further understand the need for supporting frontline staff in the provision of patient care [41]. This could lead to fostering effective and quality care [42] especially in tertiary health institutions where the workload is more pronounced [43]. In a report by USAID and HRH2030, it was revealed that the lower hospitals' annual workloads were too low, and there were unnecessary referrals to the higher hospital level [44]. This is coupled with complex cases being referred to tertiary hospitals from primary and secondary hospitals of the public and private sector [43]. It is therefore paramount to assess the influence of work-related stress on patient safety culture among nurses in a tertiary hospital.

## Materials and methods

### Study design, population of the study and sampling technique

The study adopted an analytical cross-sectional survey. An analytical cross-sectional study is a type of quantitative, non-experimental research design. These studies seek to gather data from a group of subjects at only one point in time. The purpose is to measure the association between an exposure and a disease, condition or outcome within a defined population [45]. This study was designed to assess the influence of work-related stress on patient safety culture among nurses working at Federal Medical Center Birnin-Kebbi, Kebbi State, Nigeria. The population of the study was 388 nurse employees of Federal Medical Center Birnin-Kebbi. All nurses working in the wards and units of the hospital were included in the study. Nurses with special duties outside the hospital during data collection and those on annual leave, maternity and sick leave were excluded from the study. The calculated sample size was 193, calculated using Cochran’s sample size determination formula [46]. n_0 _= Z^2^ p q / e^2^. Where no is the sample size; Z^2^ is the abscissa of the normal curve that cuts off an area α at the tails (1—α equals the desired confidence level is 95%); e is the desired level of precision; p is the estimated proportion of an attribute that is present in the population; q is 1-p.

1.96^2^ (0.5) (1–0.5)/0.05^2^ = 385. For small sample size, a correction formula is required:

*N* = n_o_ / [1 + {(n_o_ – 1) / N}]. Where n is the sample size and N is the population size = 385/ [1 + {(385–1)/388}] = 193. However, because a 20% attrition rate was added, the total number of administered questionnaires was 231, and 218 questionnaires were retrieved, yielding a response rate of 94.4%. The attrition of 5.6% was all from the non-returned questionnaires, and all efforts to retrieve them failed. After obtaining the sampling frame, the sample was selected proportionately based on the number of nurses in each ward and unit of the hospital. A systematic sampling method was used in the selection of the final sample from each ward and unit. The sampling interval was determined by dividing the total number of nurses in each ward/unit by the required nurses to be selected. Every nth number was selected, and the starting point was selected using simple random sampling.

### Study setting

The Federal Medical Center Birnin Kebbi is one of the MDAs (parastatal) under the Federal Ministry of Health. It was established under Decree 10 of 1985 i.e. the University Teaching Hospitals (Reconstitution of Boards) Act, Cap 462 on 6th June 2000. The Federal Medical Centre Birnin Kebbi took off on the site of a renovated Rural Health Centre with a seventy-five-bed capacity in 2001. The facility had been handed over to the Federal Government by the Kebbi State Government in the year 2000. Since then the Centre has grown over the years to achieve its current over three hundred-bed capacity tertiary healthcare facility amongst other milestones. The hospital is located along the Muhammad Iliyasu Bashar, Dukku Barracks road, off the ever-busy Birnin Kebbi – Jega road in the heart of the City. In 2019 the hospital recorded an average patient turnover of about one hundred and sixteen thousand, four hundred and ten patients including those from the neighbouring states and Niger Republic.

### Instruments for data collection

The instrument used for this study is made up of 3 sections as follows:

- Section A: demographic variables.

Section A constitute the demographic variables. The demographic variables assessed were age, sex, marital status and rank.

- Section B: nurses’ occupational stressor scale.

The Nurses’ Occupational Stressor Scale was adapted from Chen et al. [47]. It was a 21 items instrument with 9 subscales including work demands, work–family conflict, insufficient support from coworkers or caregivers, workplace violence and bullying, organizational issues, occupational hazards, difficulty in taking leave, powerlessness, and unmet basic physiological needs. Item 12 of the original version of the scale was removed because overtime was not part of the remuneration of civil servants in the study setting. The modified instrument for this study formed a 20-item 5-point Likert scale: Strongly Agree (SA), Agree (A), Neutral (N), Disagree (D), and Strongly Disagree (D). This was coded as 5, 4, 3, 2, and 1 for SA, A, N, D, and SD respectively. The validity of the instruments was achieved through face and content validity. Two experienced scholars in the area of the study were used to vet the instruments. The necessary corrections and advice of the scholars were effected. The pilot study was performed in a hospital different from the study setting. The respondents for the pilot study were 10% of the calculated sample size. The reliability of the instrument was determined using Cronbach’s alpha. The original scale had a reliability of 0.84 [47]. The modified form for this study had a reliability of 0.755. The measuring scales used were the mean ≤ 3.00, 3.01–4.00 and 4.01–5.00 as low, moderate and high work-related stress respectively.

- Section C: Hospital Survey on Patient Safety (HSOPS) version 2.0

The Hospital Survey on Patient Safety (HSOPS) version 2.0 was adapted from the Agency for Healthcare Research and Quality (AHRQ) [48]. The survey is having 8 subscales which include your unit/work area, your supervisor, manager, or clinical leader, communication, reporting patient safety events, patient safety rating, and your hospital. The changes made were changing the background questions to suit this study. Also, section D (Reporting Patient Safety Events) and section E (Patient Safety Rating) were not included in this study. It formed 25 items 5-point Likert scale: Strongly Agree (SA), Agree (A), Neutral (N), Disagree (D), and Strongly Disagree (D). This was coded as 5, 4, 3, 2, and 1 for SA, A, N, D, and SD respectively. The validity of the instruments was achieved through face and content validity. Two experienced scholars in the area of the study were used to vet the instruments. The pilot study was performed in a hospital different from the study setting. The necessary corrections and advice of the scholars were made. The respondents for the pilot study were 10% of the calculated sample size. The reliability of the instrument was determined using Cronbach’s alpha. The original instrument was found to have a reliability of 0.879 [49]. The modified instrument used for this study has a Cronbach’s alpha of 0.794. The measuring scales used were the mean ≤ 3.00, 3.01–4.00 and 4.01–5.00 as low, moderate and high patient safety culture respectively.

### Method of data collection and analysis

Respondents were contacted individually at their respective wards and units. This was done when the researcher visited the wards and units of the hospital to administer the instrument. Respondents were given 48 h to answer the questionnaire, after which the researcher returned and retrieved the instrument. Data generated for the study were analysed using descriptive statistics in the form of frequencies and percentages. The results are presented in tables. The inferential statistics applied were Pearson Product Moment Correlation and multiple linear regression analysis. The inferential statistic was used to ascertain the relationship between work-related stress among nurses and patient safety culture and to determine the subscales of work-related stress as predictors of patient safety culture among nurses. The Statistical Package for the Social Sciences (SPSS version 27.0) was used for data analysis.

### Ethical consideration

Ethical approval was obtained from the Federal Medical Center Birnin-Kebbi Research Ethical Committee (Number: FMC/BK/HP/015/P/517/VOLIV/053). Before administering the questionnaire, permission was obtained from the head nurse of each ward/unit. Written consent from the respondents of the study was also obtained. Confidentiality of the information provided was maintained.

## Results

Table 1 shows that respondents aged between 26 and 35 years were having the highest percentage (35.8%). The highest percentage (61.9%) of the respondents were female, while 65.1% of the respondents were married. The rank of nursing officer had the highest percentage (35.8%).

**Table 1:** Respondents’ demographic variables *N* = 218

**Variables**	**Frequency**	**Percentage**
**Age**		
≤ 25	48	22.0
26–35	78	35.8
36–45	64	29.4
> 45	28	12.8
**Sex**		
Male	83	38.1
Female	135	61.9
**Marital status**		
Single	76	34.9
Married	142	65.1
**Rank**		
NO	70	35.8
SNO	58	26.6
PNO	52	23.9
A.CNO	12	5.5
CNO	18	8.3

*Key*: *NO* Nursing officer, *SNO* Senior nursing officer, *PNO* Principal nursing officer, *ACNO* Assistant chief nursing officer, *CNO* Chief nursing officer

Table 2 shows that the age bracket ≥ 45 years had the highest percentage (50.0%) of low work-related stress. Also, female respondents had the highest percentage (45.9%) in low work-related stress than the male respondents. Married respondents had a slightly higher percentage (18.3) of work-related stress than single respondents (15.8).

**Table 2:** Association between respondents’ variables and work related stress

**Age**						
**Variables**	**≤ 25**	**26–35**		**36–45**	**≥ 45**	P^*^
Low work stress	18(37.5)	24(30.8)		26(40.6)	14(50.0)	0.44
Moderate work stress	22(45.8)	38(48.7)		30(46.9)	8(28.6)	
High work stress	8(16.7)	16(20.5)		8(12.5)	6(21.4)	
** Total**	48(100)	78(100)		64(100)	28(100)	
**Sex**						
**Variables**	**Male**			**Female**		P^*^
Low work stress	20(24.1)			62(45.9)		0.00
Moderate work stress	48(57.8)			50(37.0)		
High work stress	15(18.1)			23(17.0)		
Total	83(100)			135(100)		
**Marital status**						
**Variables**	**Single**			**Married**		P^*^
Low work stress	28(36.8)			54(38.0)		0.84
Moderate work stress	36(47.4)			62(43.7)		
High work stress	12(15.8)			26(18.3)		
Total	76(100)			142(100)		
**Rank**						
**Variables**	**NO**	**SNO**	**PNO**	**ACNO**	**CNO**	P^*^
Low work stress	32(41.0)	16(27.6)	18(34.6)	4(33.3)	12(66.7)	0.00
Moderate work stress	38(48.7)	24(41.4)	30(57.7)	4((33.3)	2(11.1)	
High work stress	8(10.3)	18(31.0)	4(7.7)	4(33.3)	4(22.2)	
Total	78(100)	58(100)	52(100)	12(100)	18(100)	

*Key*: *NO* Nursing officer, *SNO* Senior nursing officer, *PNO* Principal nursing officer, *ACNO* Assistant chief nursing officer, *CNO* Chief nursing officer

^*^Chi-square at 0.05 significant level

Table 3 indicates that the age bracket ≥ 45 had the highest percentage (57.1%) in high patient safety culture, while the ≤ 25 age bracket had the lowest percentage (12.5%). Female respondents had the highest percentage of high (28.1%) and low (9.6%) patient safety culture than the male respondents respectively.

**Table 3:** Association between respondents’ variables and patient safety culture

**Age**						
**Variables**	** ≤ 25**	**26–35**		**36–45**	** ≥ 45**	P^*^
Low patient safety culture	4(8.3)	6(7.7)		6 (9.4)	0(0.0)	0.00
Moderate patient safety culture	38(79.2)	56(71.8)		46(71.9)	12(42.9)	
High patient safety culture	6(12.5)	16(20.5)		12(18.8)	16(57.1)	
** Total**	48(100)	78(100)		64(100)	28(100)	
**Sex**						
**Variables**	**Male**			**Female**		P^*^
Low patient safety culture	3(3.6)			13(9.6)		0.01
Moderate patient safety culture	68(81.9)			84(62.2)		
High patient safety culture	12(14.5)			38(28.1)		
Total	83(100)			135(100)		
**Marital status**						
**Variables**	**Single**			**Married**		P^*^
Low patient safety culture	6(7.9)			10(7.0)		0.19
Moderate patient safety culture	58(76.3)			94(66.2)		
High patient safety culture	12(15.8)			38(40.8)		
Total	76(100)			142(100)		
**Rank**						
**Variables**	**NO**	**SNO**	**PNO**	**ACNO**	**CNO**	P^*^
Low patient safety culture	4(5.1)	6(10.3)	4(7.7)	2(16.7)	0(0.0)	0.00
Moderate patient safety culture	60(76.9)	38(65.5)	40(76.9)	8(66.6)	6(33.3)	
High patient safety culture	14(17.9)	14(24.1)	8(15.4)	2(16.7)	12(66.7)	
Total	78(100)	58(100)	52(100)	12(100)	18(100)	

*Key*: *NO* Nursing officer, *SNO* Senior nursing officer, *PNO* Principal nursing officer, *ACNO* Assistant chief nursing officer, *CNO* Chief nursing officer

^*^Chi-square at 0.05 significant level

Table 4 revealed that the highest percentage (45.0%) of the respondents experienced moderate stress. The highest percentage (69.9%) of the respondents practised a moderate safety culture, and only 22.9% practised a high safety culture.

**Table 4:** Level of work-related stress and patient safety culture among nurses *N* = 218

**Variables**	**Frequency**	**Percentage**
**Level of work-related stress**		
Low stress	82	37.6
Moderate stress	98	45.0
High stress	38	17.4
**Level of patient safety culture**		
Low culture	16	7.3
Moderate culture	152	69.7
High culture	50	22.9

Table 5 showed that there were weak or very weak significant negative correlations (*P* < 0.01) between patient safety culture practices and occupational stress across all the subscales of the nurses’ occupational stressors scale except in the occupational hazards subscale in which there was extremely weak and non-significant correlation.

**Table 5:** Correlation between patient safety culture and subscale of nurses’ occupational stressors scale

		PSC Mean	work demands Mean
PSC Mean	Pearson correlation	1	-0.227
	Significant level		0.001
		PSC Mean	work–family conflict Mean
PSC Mean	Pearson correlation	1	-0.307
	Significant level		0.000
		PSC Mean	insufficient support Mean
PSC Mean	Pearson correlation	1	-0.288
	Significant level		0.000
		PSC Mean	workplace violence Mean
PSC Mean	Pearson correlation	1	-0.252
	Significant level		0.000
		PSC Mean	organizational issues Mean
PSC Mean	Pearson correlation	1	-0.344
	Significant level		0.000
		PSC Mean	occupational hazards Mean
PSC Mean	Pearson correlation	1	-0.079
	Significant level		0.243
		PSC Mean	difficulty taking leave Mean
PSC Mean	Pearson correlation	1	-0.296
	Significant level		0.000
		PSC Mean	powerlessness Mean
PSC Mean	Pearson correlation	1	-0.151
	Significant level		0.000
		PSC Mean	unmet physiological needs Mean
PSC Mean	Pearson correlation	1	-0.284
	Significant level		0.000

Pearson product moment correlation

Table 6 indicated that the overall usefulness of the model in explaining the patient safety culture is significant, *F* (9, 208) = 6.093, *p* < 0.05. Work–family conflict was a significant predictor of patient safety culture, t (208) = -2.341, *P* < 0.05. Workplace violence and bullying was a significant predictor of patient safety culture, t (208) = -1.984, *P* < 0.05. Occupational hazards was a significant predictor of patient safety culture, t (208) = 2.446, *P* < 0.05. Difficulty in taking leave was a significant predictor of patient safety culture, t (208) = -2.190, *P* < 0.05.

**Table 6:** Occupational stressor scale subscales as determinant of patient safety culture

**Occupational stressor scale subscales**	**coefficient**	**standard error**	**t**	P^*^
Work demands	0.001	0.039	0.018	0.99
Work–family conflict	-0.103	0.044	-2.341	0.02
Insufficient support from coworkers	0.001	0.046	0.025	0.98
Workplace violence and bullying	-0.053	0.027	-1.984	0.05
Organizational issues	-0.050	0.037	-1.340	0.18
Occupational hazards	0.106	0.043	2.446	0.02
Difficulty in taking leave	-0.062	0.028	-2.190	0.03
Powerlessness	0.041	0.043	0.941	0.35
Unmet basic physiological needs	-0.072	0.038	-1.884	0.06

R^2^ = 0.209

F ratio = 6.093 *P* < 0.05

Standard error of the estimate = 0.415

*N* = 218

^*^Multiple linear regression

## Discussion

This study aimed to assess the influence of work-related stress on patient safety culture among nurses in a tertiary hospital. The findings revealed that the highest percentage (over two-fifths) of nurses had moderate perceived work-related stress. Additionally, nearly four–fifths of the respondents perceived moderate patient safety culture. A moderate level of work-related stress corresponded with a moderate level of patient safety culture. The literature has shown that work-related stress affects the caring behaviour of nurses due to overactivity and high workload [16]. This finding is crucial in planning an intervention toward reducing the incidence of breaches in patient safety, which is described by the WHO as a critical global public health issue and has a widely accepted role in enabling health systems to achieve effective Universal Health Coverage (UHC) [50].

It seems that nurses mostly experienced work-related stress at the level of moderate to high. This is found in other studies such as a cross-sectional study on occupational stress experienced by nurses working in a Greek Regional Hospital found nurses to have experienced a moderate level of work-related stress [22]. Additionally, in a study in Nigeria, 80.5% of nurses were revealed to have moderate job stress [23]. A study in Ghana on psychological working conditions and predictors of occupational stress among nurses, Salaga Government Hospital, found 21.0% of the respondents’ level of stress to be between high and extreme levels [51]. It is seldom to find a study with the majority of nurses experiencing a low level of work-related stress. The implication of these findings is the need for an intervention to reduce the level of work-related stress experienced by nurses and in turn, boost the level of patient safety.

The moderate to high level of occupational work-related stress associated with the nursing profession is linked to the overwhelming responsibility of nurses as patient safety keepers, care and treatment givers, catalysers of patient recovery, providers of palliative care and many more. However, Veda and Roy [3] postulated that nurses often consider the quality of care to patients, ignoring their healthy well-being. Ideally, workers’ health is among the crucial factors to be considered if safety care is the priority [52]. The healthy well-being of nurses is considered important not only to nurses themselves but also to patients. In essence, stressful situations can cause damage to the mental ability of the affected individual, resulting in low job productivity [14, 15]. Nurses with a moderate to high level of stress can face mental distress leading to anxiety and depression, which could negatively affect nursing care.

The findings of this study also revealed that nurses had a moderate patient safety culture. The level of stress aligns with the level of patient safety. This finding aligns with a study in Ethiopia, where it was found that less than half of the respondents perceived patient safety culture to be good [53]. Thus, there is a need for measures aimed at improving healthcare safety. These measures include interventions to reduce work-related stress. To improve the quality of care, acknowledging improved safety culture by healthcare organisations is becoming more pertinent [54]. Improving safety culture among nurses is an inevitable key to ensuring patient safety. It is asserted that observing standards in safety culture during every day patients’ treatments can be used to lessen treatments’ adverse events and damages [55]. In a nutshell, reducing work-related stress could lead to increased patient safety culture and quality of care.

The results of this study revealed that there were significant negative correlations between all subscales of work-related stress and safety culture practice among nurses except occupational hazards subscale in which the strength of the correlation was extremely weak and non-significant. This is an important indication of the influence of work-related stress on patient safety culture, even though the correlations were mostly weak or very weak. The correlations were found to be weak or very weak, but the effect it could lead to should not be taken lightly. This finding is in accordance with a finding of a study by Babapour et al. [18] where it was found that the total score of caring behaviours had a negative correlation with the total and component scores of job stress. According to the authors [18], caring behaviour or safety culture decreases with increased job- or work-related stress. This means that the consequences of work-related stress among nurses affect the functioning of individual nurses and the organisation at large [22]. The professional attitude of nurses is therefore affected by moderate to high levels of stress. The work-related stress among nurses predisposes them to poor professional communication, leading to lower-quality nursing care [2]. Thus, work-related stress influences professional attitudes, which in turn affects individual nurses and organisational functioning, leading to a breach in patient safety culture.

The work-related stress is said to be related to individual characteristics such as age and gender [56]. In the results of this study, there was an element of impact of age on work-related stress. This is shown as the highest age bracket constituted the highest percentage with low work-related stress level. This is also supported by the assertion that age is positively associated with the experience and work stress management ability is found to be increasing with an increase in age [57]. Workers whose age is less than 40 years are most likely to experience high-stress levels [58]. This could be associated with the ability to cope with the stress. Personal characteristics like age, gender and family condition have a significant effect on stress-coping ability [59, 60]. However, the result of this study has shown no significant association between age and work-related stress. Sabzi et al. [61] and Romano et al. [62] also had the same findings. Other factors such as coping ability, state of employee career development and job satisfaction could have been mediating factors between work-related stress and employees’ age.

Though Faraji et al. [58] opined that occupational stress equally affects both males and females, the findings of this study revealed the contrary. Female respondents recorded the highest percentage in low work-related stress, while male respondents had their highest percentage in moderate work-related stress. Thus, male respondents experienced higher work-related stress than female respondents. The finding revealed a significant association between gender and work-related stress in this study. Also, contrary to the findings of this study, other studies found females to have experienced higher levels of wok-related stress than males [9, 63, 64]. The difference could be due to differences in study areas, scoring system and most likely the sample size. Babapour et al. [18] attributed the lack of significant difference between male and female respondents in work-related stress to be due to a larger number of female respondents compared to males. However, this study contradicts this assertion in which the female respondents were more than the males, but still significant association was found.

In this study, the married respondents had slightly higher work stress than single respondents with no significant association. In consonance with this study other studies [2, 18] found no significant difference between marital status and stress level. However, the authors asserted that life issues associated with married individuals could have an impact on work-related stress [18]. Contrary to these findings, Nyamwata et al. found a significant association between marital status and work-related stress. The authors opined that marriage life could be a source of stress serving as a synergy to wok-related stress [65]. Ghafoor et al. in their study concluded that workers who are married encounter more stress than unmarried workers [66]. The most important likely reason for the differences was the circumstances of marriage life. Different couples have different marriage life circumstances. Thus, the wok-elated stress could be affected by the quality of the marriage between the couples [57].

There was an element of association between the respondents' rank and work-related stress. The highest rank was shown to have a higher percentage of low-stress level. Also, the rank with the lowest work-related stress level was principal nursing officer. The lower-ranked nurses were more likely to be stressed than the higher-ranked nurses. Osei-Mireku et al. postulated that the higher the experience of an employee the lower the stress they experienced [57]. This may be connected with the ability of highly experienced employees (who are mostly high-ranked) to delegate duties. Moreover, age is positively correlated with the ability to handle stress; and experience (rank) is accompanied by age [63]. Nurses with lower years of service are more likely to be stressed than nurses with higher years of service [9]. The senior nurses have gotten enough experience in death and dying, and problem solving. The experiences of senior nurses reduce the magnitude effect of the stress they are exposed to.

The results of this study revealed a significant association between the respondents’ age and patient safety culture. The highest age bracket had the highest percentage in high patient safety practice and the lowest percentage in low patient safety practice. In accordance with this finding Kakemam et al. asserted that older nurses’ patient safety is better than younger ones [67]. The age of the respondents could therefore be a factor that increases the practice of patient safety practices among nurses. There is a postulation that nurses aged within the 40–60 years age bracket had a better understanding of patient safety [68], and therefore could have an influence on the culture of patient safety. Moreover, in this study, there was a significant association between gender and patient safety culture. Female respondents had the highest percentage in high and low patient safety culture levels respectively. This is in accordance with the fact that female respondents had the highest percentage in low work-related stress level than male respondents. The work-related stress was negatively correlated with patient safety culture. In accordance with this finding another study found that patient safety culture was significantly associated with sex [69].

Married respondents in this study were found to have a higher percentage in high patient safety culture and a lower percentage in low patient safety culture. This means that married respondents had a better patient safety culture. However, there was no significant association between marital status and patient safety culture. Contrary to this finding Morika et al. [70] found unmarried nurses to have a higher percentage in implementing patient safety compared to married nurses. But in accordance with this study the authors found no significant relationship between marital status and implementing patient safety among nurses. Also, in accordance with the finding of this study, a study by Shashamo et al. found no significant association between marital status and patient safety culture [69]. However, Salih et al. found a significant association between nurses’ attitudes to patient safety and marital status [71]. This difference with regard to Salih et al. study could be from differences in variable categorization, as well as differences in the instrument used. Nevertheless, Marriage life is more likely to stabilise an individual and lead to effective practice. Employees who are married are more satisfied with their job and have lower turnover than their unmarried counterparts [70].

In this study, the highest-ranked nurses were found to have the highest percentage in high patient safety culture and the lowest percentage in low patient safety culture with a strong significant difference. This revealed the possibility of an influence of nurses’ rank on patient safety culture. The higher the rank the higher the patient safety culture. This is in accordance with the assertion that age is positively correlated with the ability to handle stress, and experience (rank) accompanied age [63]. Thus, the higher the rank the more possibility of appropriate work-related stress management and in turn more effective patient safety. Caring behaviour or safety culture among nurses decreases with an increase in job- or work-related stress [18]. As high-rank nurses are more likely to adequately manage work-related stress, they are also more likely to exhibit a positive patient safety culture. This called for the need for intensifying supervision from experienced senior nurses to less experienced junior nurses. The most likely result of this is improved patient safety.

Five (work–family conflict, workplace violence and bullying, organizational issues, difficulty in taking leave, and unmet basic physiological needs) out of 9 occupational stressors were found to have a negative prediction on patient safety culture. Thus, as these variables increased the patient safety culture decreased. With 1 unit increase in work-family conflict, the patient safety culture decreased by -0.103. With 1 unit increase in workplace violence and bullying, the patient safety culture decreased by -0.053. With one unit increase in organizational issues, the patient safety culture decreased by -0.050. With 1 unit increased in difficulty in taking leave, the patient safety culture decreased by -0.062. With 1 unit increase in unmet basic physiological needs, the patient safety culture decreased by -0.072. This finding is in accordance with a study conducted in Venezuela in which occupational factors were found to be the primary predictors of the patient safety culture index [72]. Another study by Ismail et al. [73] revealed that the effect of demanding or stressful conditions on patient safety impairs the provision of safe and effective treatment.

However, the finding of this study indicated 4 of the occupational stressors have had positive predictions on the patient safety culture. Nevertheless, the positive predictions they had were extremely weak (coefficients of 0.001, 0.001, and 0.041) which could be considered as negligible, except the occupational hazards stressor with 0.106 which could be considered as weak. It is not clear why the occupational hazards variable was not among the variables with a negative prediction on patient safety culture. This is because workers' safety mostly positively correlates with patient safety [74]. Even though there is virtually no study that looks into the relatedness of the health care workers' occupational hazards and patient safety culture, the possible explanation for this finding is the assertion by Veda and Roy [3] that in considering the quality of care, nurses often disregard their well-being. Thus, nurses could not have considered occupational hazards as a factor that could affect their patient safety culture because of their keen need for the safety of the patient.

The methodological limitations of this study include dependence on respondents’ judgement in assessing work-related stress and patient safety practice. Depending on respondents’ judgement is associated with biases from the respondents. However, the method applied is the most feasible since making an observational study is out of the capability of the authors. The inability to get a 100% response rate despite the added attrition rate is another important limitation. Nevertheless, the study provides an impetus towards understanding the subject matter in the study area. However, the study is generalizable to the population of the study because of the rigorous systematic sampling procedure followed in selecting the sample. Also, the response rate was above the required sample size initially calculated before adding the attrition rate. This makes the sample size a little bit larger.

## Conclusion

Work-related stress is implicated as one of the major factors leading to reduced patient safety culture. Chronic work-related stress among nurses is a precursor for a breach in patient safety culture and quality of nursing care. Therefore, understanding the factors necessary for mitigating work-related stress among nurses is of great use in giving support to the nurses who are at the frontline in giving safe care to patients. Thus, for nurses to provide effective and quality nursing care that considers a patient safety culture, differences in perceived stress among nurses’ age, gender, marital status and rank should be considered in allocating duties and providing necessary assistance. This study’s findings imply that the improving supervision process through which the senior nurses supervise the younger ones could be of great importance in reducing stress among junior nurses. There should be a policy prohibiting junior nurses from working alone without supervision. This would help reduce stress and improve patient safety.

## Data Availability

The datasets used or analysed during the present study are available from the corresponding author upon reasonable request. The data are not publicly available due to privacy and ethical restrictions.

## Abbreviations

AHRQ: Agency for Healthcare Research and Quality
UHC: Universal Health Coverage
